# Comparison of Slides and Video Clips as Different Methods for Inducing Emotions: An Electroencephalographic Alpha Modulation Study

**DOI:** 10.3389/fnhum.2022.901422

**Published:** 2022-06-06

**Authors:** Zaira Romeo, Francesca Fusina, Luca Semenzato, Mario Bonato, Alessandro Angrilli, Chiara Spironelli

**Affiliations:** ^1^Department of General Psychology, University of Padova, Padua, Italy; ^2^Padova Neuroscience Center, University of Padova, Padua, Italy

**Keywords:** emotional processing, EEG source localization, emotional pictures, movies clips, valence, arousal, sLORETA, Mu rhythm

## Abstract

Films, compared with emotional static pictures, represent true-to-life dynamic stimuli that are both ecological and effective in inducing an emotional response given the involvement of multimodal stimulation (i.e., visual and auditory systems). We hypothesized that a direct comparison between the two methods would have shown greater efficacy of movies, compared to standardized slides, in eliciting emotions at both subjective and neurophysiological levels. To this end, we compared these two methods of emotional stimulation in a group of 40 young adults (20 females). Electroencephalographic (EEG) Alpha rhythm (8–12 Hz) was recorded from 64 scalp sites while participants watched (in counterbalanced order across participants) two separate blocks of 45 slides and 45 clips. Each block included three groups of 15 validated stimuli classified as Erotic, Neutral and Fear content. Greater self-perceived arousal was found after the presentation of Fear and Erotic video clips compared with the same slide categories. sLORETA analysis showed a different lateralization pattern: slides induced decreased Alpha power (greater activation) in the left secondary visual area (Brodmann Area, BA, 18) to Erotic and Fear compared with the Neutral stimuli. Instead, video clips elicited reduced Alpha in the homologous right secondary visual area (BA 18) again to both Erotic and Fear contents compared with Neutral ones. Comparison of emotional stimuli showed smaller Alpha power to Erotic than to Fear stimuli in the left precuneus/posterior cingulate cortex (BA 7/31) for the slide condition, and in the left superior parietal lobule (BA 7) for the clip condition. This result matched the parallel analysis of the overlapped Mu rhythm (corresponding to the upper Alpha band) and can be interpreted as Mu/Alpha EEG suppression elicited by greater motor action tendency to Erotic (approach motivation) compared to Fear (withdrawal motivation) stimuli. Correlation analysis found lower Alpha in the left middle temporal gyrus (BA 21) associated with greater pleasantness to Erotic slides (*r*_38_ = –0.62, *p* = 0.009), whereas lower Alpha in the right supramarginal/angular gyrus (BA 40/39) was associated with greater pleasantness to Neutral clips (*r*_38_ = –0.69, *p* = 0.012). Results point to stronger emotion elicitation of movies vs. slides, but also to a specific involvement of the two hemispheres during emotional processing of slides vs. video clips, with a shift from the left to the right associative visual areas.

## Introduction

The processing of emotional information plays a central role in human cognition and social interactions. Experiencing emotion involves different components, including physiological reactions, mental states and explicit behaviors (Matsumoto and Hwang, 2012). Specific emotional experiences (e.g., positive or negative states) can modify our cognitive functioning by altering decision making, attentional, learning, mnestic and perceptual processes (Phelps, 2004; Vuilleumier, 2005; Lerner et al., 2015; Tyng et al., 2017). Given the multicomponential nature of emotion, the implementation of experimental paradigms for assessing both behavioral and neural responses remains challenging. A critical issue concerns the type of stimuli used for eliciting emotional responses (Gross and Levenson, 1995). Many studies have been using standardized databases (e.g., IAPS; Lang et al., 1997), consisting in pleasant and unpleasant static pictures. However, dynamic stimuli, such as short videoclips, have proven to be among the most effective techniques for studying emotional processing in the laboratory (Rottenberg et al., 2007). Dynamic materials are more ecological because they present more information from various sensory inputs (i.e., visual and auditory information). All these characteristics make dynamic stimuli both more similar to reality and more effective in inducing complex emotional scenarios. Their superiority has been widely documented in literature since the ‘90s. For example, a meta-analysis by Westermann et al. (1996) compared film clips with ten other emotion induction techniques and found them to outperform all alternative methods in eliciting both positive and negative emotional states (Westermann et al., 1996). More recently, McGinley and Friedman (2017) compared imagery, recall and film clip viewing and found the latter to be the best-performing technique in the prediction of emotion-specific patterns of autonomic nervous system activation, with 4 out of the 5 emotional states considered in the study being correctly identified (McGinley and Friedman, 2017). Moreover, the development of emotional states, especially if complex, has been shown to need time to develop (Rottenberg et al., 2007). While a movie clip, even if realistic, is not the same as a real-life situation, these findings point to dynamic stimuli as the best compromise between exerting a necessary degree of control over experimental variables and allowing for a sufficiently immersive emotional experience for the viewer (Rottenberg et al., 2007). Surely, imagery of personal past experiences is a very effective method for inducing emotions, yet it allows virtually no control on what the participant is really imaging. Another advantage of using film clips derives from the fact that, typically, film makers induce viewers to empathize with characters from very early on in the film by using various visual techniques, such as shallow focus, close-ups, etc. (Plantinga et al., 1999; Coplan, 2006). Watching a movie elicits a complex combination of passive viewing and imaginative processes, which involve both cognition and affect, and consist in adopting a character’s emotional state and cognitive point of view. This is elicited to boost the communicative efficacy of movies (Coplan, 2006). While movies achieve this emotional contagion via long durations, even short clips may trigger an empathic response, especially in individuals with high levels of trait empathy. During the last years, both static and dynamic settings have been used for investigating the brain signatures of positive and negative emotional content. A shared view is that emotional processing involves a rather complex neural circuitry which includes the amygdala, the anterior cingulate, the temporal lobe, the insular cortex, the prefrontal cortex and the visual areas (Davidson and Irwin, 1999; Lane and Nadel, 2002; Phan et al., 2002). Concerning emotional valence (i.e., pleasant and unpleasant stimuli), it has been proposed that the ventro-medial portion of the prefrontal cortex participates in the general representation of basic emotional states (both positive and negative), whereas the amygdala is particularly activated by negative affect (e.g., fear) (Davidson and Irwin, 1999). In contrast, pleasant pictures mainly recruit the basal ganglia, including the putamen and the ventral striatum (Davidson and Irwin, 1999; Phan et al., 2002). In addition to these results, other hypotheses suggest different hemispheric contributions to the emotional experience. According to Davidson (1995, 1992), the two hemispheres are involved in emotional processing in different ways: the right hemisphere is activated by negative affect, whereas the left hemisphere by positive affect. Another influential theory supports right hemisphere dominance in the processing of all emotions, regardless of their positive or negative valence (Borod et al., 1998). Both these models were confirmed by studies on healthy participants and brain-damaged patients, thus suggesting a possible coexistence of the two mechanisms (Borod et al., 1998; Killgore and Yurgelun-Todd, 2007; Prete et al., 2015). However, the heterogeneity of the results leaves many questions open and other approaches could be considered for the investigation of the neural correlates of emotions. For example, recent neuroimaging works propose new analysis methods with a focus on whole-brain network dynamics (Barrett and Satpute, 2019). From an electrophysiological perspective, the Electroencephalographic (EEG) Alpha band (8–12 Hz) has been widely investigated as a potential marker of emotional processing. The interest for this specific rhythm is partially due to Alpha activity being inversely correlated with cortical brain activity (Lindsley and Wicke, 1974) and, thus, representing an index of cortical inhibition. A large body of studies tested the hypothesis of frontal Alpha asymmetry during the processing of different emotional materials (for a review, see Harmon-Jones, 2003). More specifically, unbalanced Alpha distribution within the left and right frontal areas was associated with approach-avoidance processes, with less left vs. right Alpha for approaching and less right vs. left Alpha for avoiding behaviors (Coan and Allen, 2004; Harmon-Jones et al., 2010). However, it is still not clear whether this asymmetric pattern is the result of greater activity on one hemisphere or, on the contrary, of pronounced inhibition on the other (Uusberg et al., 2013). Furthermore, this literature studied the correlation between electrophysiological measures (and Alpha waves in particular) collected at rest, and emotion assessed with personality trait and behavior attitude questionnaires. This approach is rather different from the direct study of the electrophysiological correlates of emotional processing in a visual task. Focusing on the Alpha-emotions relation, past studies are not consistent. For instance, some authors reported increased Alpha power for emotional compared with neutral stimuli both in anterior and posterior regions (Aftanas et al., 2002, 2004), while others found Alpha decreasing during emotional conditions, both in anterior and posterior sites (Everhart and Demaree, 2003; Sarlo et al., 2005; de Cesarei and Codispoti, 2011; Schubring and Schupp, 2019). Notably, some studies did not find any relation between Alpha rhythm and emotional processing (Müller et al., 1999; Balconi and Lucchiari, 2006). A possible explanation for these heterogenous results could be related to the different emotional elicitation methods adopted in these studies, including affective word lists, pictures from various databases and clips. As mentioned above, dynamic emotion induction methods (e.g., movie clips) overcome several limitations presented by pictures (Westermann et al., 1996). In view of that, using dynamic and more ecological stimuli for inducing emotional reactions could also allow to better frame the neural underpinnings of emotions. In particular, whether static and dynamic scenarios produce similar or different Alpha modulation is still unclear. On one hand, it could be expected that emotional clips enhance the activation of the same circuits involved in the processing of slides. On the other hand, clips could require the contribution of different networks/hemispheres given the engagement of multimodal stimulation that need to be integrated to form a unitary, coherent emotional representation. To test these hypotheses, we carried out an EEG experiment in a selected group of young adults. We controlled the potential influence of individual characteristics on emotional responses by matching our participants for age, education levels, gender, and empathic traits. A previous study showed differences in the affective responses and electrophysiological activation during emotional clip viewing in women with low vs. high trait empathy (Maffei et al., 2019). The emotional stimuli used in the present study consisted in both static slides and brief, dynamic movie clips, having pleasant (Erotic) and unpleasant (Fear) valence. In addition, neutral stimuli were presented as a control condition. In order to make slides and clips comparable, all stimuli were presented for 13 s. Subjective measures of stimulus valence and arousal were also collected to measure the effect of our emotional manipulations (static vs. dynamic). EEG source estimation on the Alpha rhythm was data-driven and computed to investigate brain reactivity to positive and negative emotions during slide and clip presentations. Overall, our experimental design allowed assessing: (I) the main hypothesis of a greater effectiveness of dynamic (clips) vs. static (slides) stimuli in capturing emotional responses; (II) the EEG hemispheric distribution during pleasant and unpleasant vs. neutral emotional processing; (III) the Alpha modulation induced by slides vs. clips.

## Materials and Methods

### Participants

A preliminary online questionnaire was completed by a sample of 264 young adults. This form allowed us to select the final sample according with our inclusion criteria. In particular, only heterosexual participants were enrolled in the study, due to the content of our erotic emotional stimuli, which were characterized by the presence of heterosexual couples only. We then excluded participants with specific phobias (e.g., phobia for firearms, knives, blood) and/or with neurological or psychiatric disorders. The final sample was thus composed by 40 participants (20 women, mean age: 22.92 years, ± 2.69) with normal or corrected-to-normal vision. All participants were more than 18 years old and gave their written informed consent to take part in the experiment, according to the Declaration of Helsinki. The experimental procedure was approved by the Psychology Ethics Committee of the University of Padova (Protocol n. 3886).

### Behavioral Scales

A series of questionnaires were administered to measure some psychological characteristics of our sample. For the assessment of empathic traits, all participants completed the Interpersonal Reactivity Index (IRI) questionnaire, consisting in 4 subscales: *Empathic Concern*, *Personal Distress*, *Fantasy* and *Perspective Taking* (Davis, 1983). More in detail, *Empathic Concern* refers to feeling sympathy for unfortunate others; *Personal Distress* assesses the stress the participant may feel when experiencing a difficult interpersonal situation; *Fantasy* analyzes the ability of the participant to self-identify with fictional characters; and *Perspective Taking* concerns how much the participant tends to spontaneously adopt the point of view of others. A separate score was computed for each subscale, and their sum contributed to the total IRI score. The presence of phobias was measured using an *ad hoc* short Italian version of the Fear Survey (Wolpe and Lang, 1964), focused on situations or things that may cause unpleasant feelings (e.g., animals, social stimuli, tissue damage such as injections, or illness). Both measures were collected during the online screening phase. The day of the experiment, participants were asked to complete three additional questionnaires: the State-Trait Anxiety Inventory Y (STAI-Y) 1 and 2, measuring both state and trait anxiety (Spielberger et al., 1983) and the Positive and Negative Affect Schedule (PANAS), measuring the participants’ positive and negative feelings (Watson et al., 1988). The PANAS was also administered at the end of the EEG session for monitoring potential mood changes following the experimental manipulation.

### Experimental Setting

The experiment was carried out in a dedicated EEG laboratory. Since EEG data were collected during the COVID-19 pandemic, participants were requested to wear face masks during the whole experimental setting. Experimenters wore face masks with eye shields, and gloves during the procedure that allowed them to prepare each participant for EEG recording.

**FIGURE 1:**
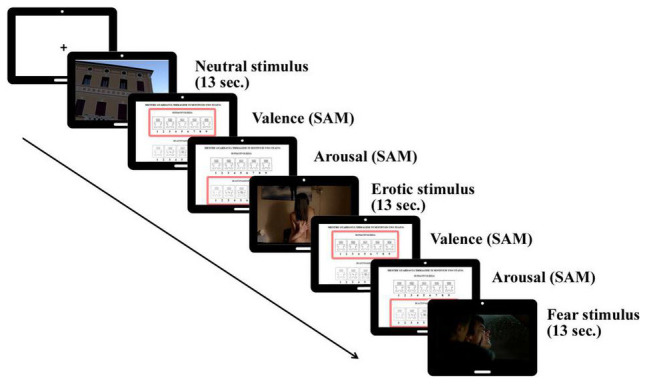
Trial sequence of passive viewing of neutral, erotic and fear stimuli (slides/clips), followed by SAM (Self-Assessment Manikin) judgments (valence/arousal).

The EEG session included a passive viewing condition consisting in the presentation of static and dynamic stimuli from two categories: neutral stimuli (e.g., scenes representing landscapes, documentaries, or daily activities) and emotional stimuli, which could be pleasant (i.e., erotic scenes) or unpleasant (i.e., fear scenes). The static condition consisted in the presentation of 45 still slides (15 neutral, 15 erotic and 15 fear), whereas the dynamic condition consisted in the presentation of 45 short clips (15 neutral, 15 erotic and 15 fear) from commercial movies or documentaries. The slides represented a screenshot of each clip—this was done in order to achieve comparable levels of the visual components of the stimuli (e.g., brightness, contrast, color levels) in both conditions. Both slides and clips were presented for 13 s to keep the stimuli duration constant in the two conditions (static vs. dynamic). After each image/clip appearance, participants were asked to judge the valence and the arousal elicited by each stimulus using the Self-Assessment Manikin (SAM; Bradley and Lang, 1994) by typing on a numeric keypad a value ranging from 1 (low valence/arousal) to 9 (high valence/arousal). After the participants gave their rating, the next stimulus appeared on the screen (see Figure 1). Notably, while no sounds were presented during the presentation of the static slides, in the emotional (i.e., erotic and fear) clips we kept the original audio, while neutral clips had a musical score only. The static and dynamic conditions were counterbalanced: participants 1–20 first viewed the static slides and then the dynamic clips, while participants 21–40 first viewed the dynamic clips and then the static slides.

### Electroencephalographic Data Recording and Preprocessing

EEG data were collected using a standard 64 channels cap (Acticap, BrainProduct system). EEG was recorded in DC mode and the activity of each electrode was online referred to FCz. The sampling rate was set at 1,000 Hz and the impedance was kept below 5 kΩ throughout the recording. The activity of FCz was reconstructed and data were off-line re-referenced to the average reference. Bad channels were interpolated with the triangulation and linear interpolation method using the BrainVision Analyzer software (Brain Products GmbH, Germany). Signal processing was then carried out using Brainstorm toolbox (Tadel et al., 2019). Data were filtered using a band-pass filter (lower cutoff = 0.5 Hz; upper cutoff = 125 Hz; stop-band attenuation = 60 dB). An Independent Component Analysis (ICA; method = Infomax EEGLAB/RunICA) was applied on the filtered data to identify ocular artifactual components. Components associated with eye artifacts (i.e., blinks, horizontal and vertical eye movements) were detected and then removed. Artifact-free data were then segmented in epochs of 2 s. Epochs with residual noise were deleted first by using a peak-to-peak procedure (threshold value = ± 150 μV) that rejected the entire epoch, and lastly by visually inspecting the residual artifact-free epochs.

### Behavioral Analyses

Behavioral data were analyzed using a 3 (Stimulus content: Neutral vs. Erotic vs. Fear) x 2 (Condition: slides vs. clips) within-subject ANOVA, both for the Valence and Arousal self-reports. Data analyses were performed using the Statistica 6.1 (StatSoft GmbH) software. *Post hoc* comparisons were computed using the Newman-Keuls method (*p* < 0.05), and the Greenhouse-Geisser correction was applied when necessary (df > 2). In addition, we carried out behavioral correlations between SAM indices and the psychological traits that were measured with the STAI, PANAS and IRI questionnaires.

### EEG Source Analyses

Localization of the neural sources underlying the effects of the emotional stimulation in the static and dynamic conditions was computed using the standardized low resolution brain electromagnetic tomography (sLORETA) method (Pascual-Marqui, 2002). sLORETA estimates the smoothest possible 3D distributed source density solution in gray matter after 5,000 permutations. Starting from all epochs available from each participant and category, a single, 64 × 64 complex-valued, cross-spectral matrix for each participant and condition was computed for the Alpha frequency range (8–12 Hz). In addition, as we used fear images/clips strongly related to action/movement (namely fear, but also erotic induce appetitive action tendency, that is approach toward the pleasant stimulus), the Mu rhythm (10–12 Hz), typically associated with movement planning/execution (e.g., Garcia-Rill, 2015), has been further analyzed to test whether stimuli involving movement elicited particular activation, compared to those (i.e., neutral) that did not involve movement. All cross-spectral matrices were then converted in sLORETA transformation matrices to reduce noise associated with measurement, to minimize the dependence of the source current density on individual subjects, and to eliminate components in the EEG spectra that were common to both groups. In the analysis step corresponding to the application of the transformation matrix to the original electrode coordinates, we followed Pascual-Marqui’s indications (2002), which recommended to use the standard setting without selecting a specific regularization parameter alpha (option “none”). This transformation algorithm uses the three-shell spherical head model registered to the Talairach Human Brain Atlas (Talairach and Tournoux, 1988) available as MNI coordinates.

We investigated the effect of our experimental manipulation (slides vs. clips presentation) and also contrasted emotional and neutral stimuli. In particular, we compared the electrophysiological activity in neutral and fear, neutral and erotic and fear and erotic stimuli both for static and dynamic conditions (two-tailed *t*-tests). To further investigate the association between the neural responses to emotional stimuli and the individual emotional state (explicit valence and arousal ratings), we computed sLORETA correlation analyses between the Alpha rhythm and SAM scores. These analyses were performed for each experimental condition (slides vs. clips) and for each stimulus category (neutral, erotic and fear). sLORETA measures were also correlated with the scores obtained by the participants at the STAI, PANAS and IRI tests. All results are reported in MNI coordinates.

## Results

### Behavioral Data

**FIGURE 2:**
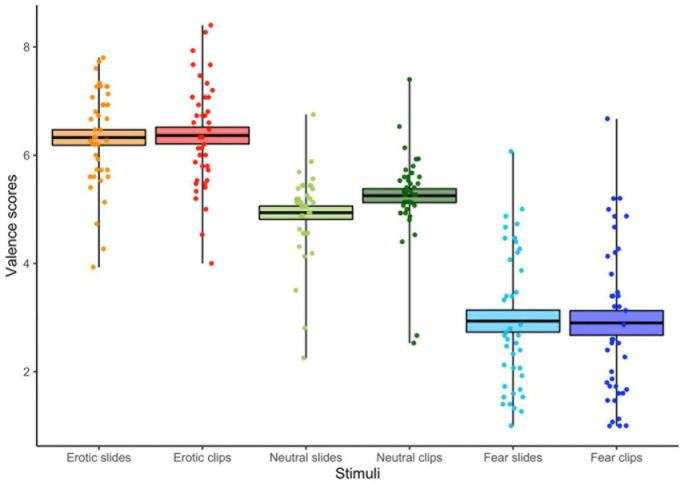
Valence scores for each condition (slides vs. clips) and stimulus content (erotic vs. neutral vs. fear). The horizontal black lines indicate the mean and colored areas indicate Standard Errors (SE). Dots represent the individual responses.

The ANOVA (3 Stimulus content [Erotic vs. Neutral vs. Fear] X 2 Conditions [Slides vs. Clips]) carried out on the valence scores showed a main effect of Stimulus content [*F*_(2,78)_ = 126.76, *p* < 0.001, GG ε = 0.78, η^2^*_*p*_* = 0.76] revealing greater pleasantness to erotic than to both neutral and fear stimuli, and greater unpleasantness to fear stimuli compared to both neutral and erotic stimuli, regardless of the kind of presentation (slides vs. clips). Our analysis also revealed an interaction between the condition and the stimulus content [*F*_(2,78)_ = 2.97, *p* = 0.05, GG ε = 0.72, η^2^*_*p*_* = 0.07]. In particular, as shown in Figure 2, neutral clips were preferred to static slides, while both emotional stimuli elicited similar valence rates during slides and clips presentations.

**FIGURE 3:**
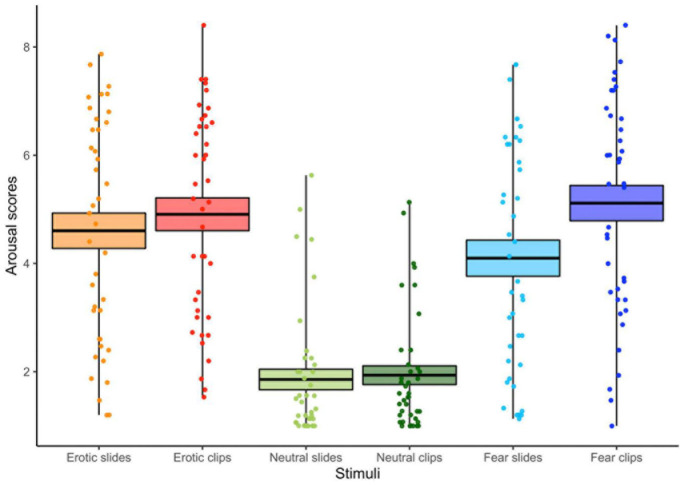
Arousal scores for each condition (slides vs. clips) and stimulus content (erotic vs. neutral vs. fear). The horizontal black lines indicate the mean and colored areas indicate Standard Errors (SE). Dots represent the individual responses.

The ANOVA (3 Stimulus content [Erotic vs. Neutral vs. Fear] X 2 Conditions [Slides vs. Clips]) computed on the arousal scores showed main effects for Condition and Stimulus content. Greater arousal was associated to clips vs. slides, confirming the effectiveness of our experimental manipulation [*F*_(1, 39)_ = 18.48, *p* < 0.001, η^2^*_*p*_* = 0.32]. Moreover, arousal was significantly higher for the two emotional stimuli compared with the neutral ones [*F*_(2, 78)_ = 75.12, *p* < 0.001, GG ε = 0.93, η^2^*_*p*_* = 0.66]. A condition x stimulus interaction was also found [*F*_(2, 78)_ = 20.41, *p* < 0.001, GG ε = 0.97, η^2^*_*p*_* = 0.34] (see Figure 3). Viewing of neutral slides and clips resulted in similar arousal levels. However, emotional clips increased the arousal compared to still slides (all *p*s < 0.01). Notably, the arousal score for erotic slides was significantly higher than for fear slides.

Correlations between the SAM and psychological traits showed two negative associations between valence scores for the unpleasant clips (fear) and both the Perspective Taking subscale (*r* = –0.34) and the total score of IRI (*r* = –0.38). These results suggest that participants with higher general empathy and/or higher capability to spontaneously adopt the point of view of others provided lower valence ratings to fear clips.

### EEG Source Localization

We report below the results of the comparisons between different conditions for the Alpha EEG rhythm (8–12 Hz) as well as the Mu activity (10–12 Hz).

#### Neutral vs. Fear Stimuli–Slides and Clips Conditions

**FIGURE 4:**
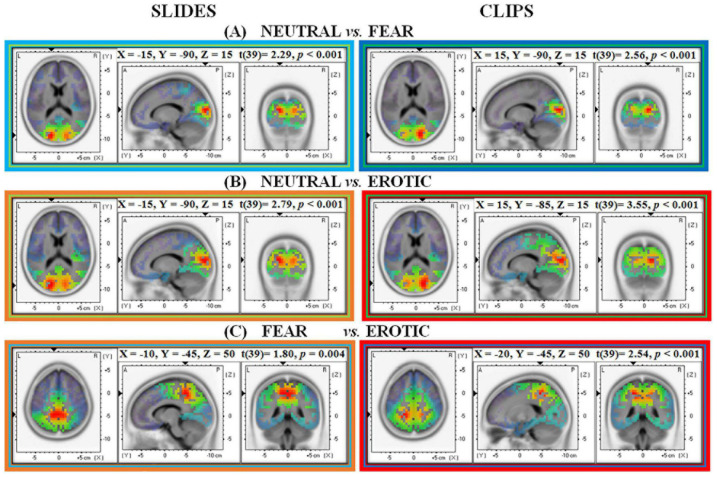
Neural source estimation for pictures and clips conditions in Alpha EEG frequency range (8–12 Hz). Each panel shows a different contrast: **(A)** Neutral vs. Fear; **(B)** Neutral vs. Erotic; **(C)** Fear vs. Erotic.

For slides source analysis revealed greater Alpha activity in the left hemisphere secondary visual cortex (MNI: X = –15, Y = –90, Z = 15; Broadmann area 18) during neutral than fear slides presentation (*t* = 0.229, *p* < 0.001). In contrast, for neutral vs. fear clips (*t* = 0.256, *p* < 0.001) greater Alpha was found in the homologous right hemisphere secondary visual cortex (MNI: X = 15, Y = –90, Z = 15; Broadmann area 18) (see Figure 4A).

The further analysis of Mu rhythm revealed the same MNI coordinates for both slides (MNI: X = –15, Y = –90, Z = 15; Broadmann area 18; *t* = 0.18, *p* < 0.001) and clips (MNI: X = 15, Y = –90, Z = 15; Broadmann area 18; *t* = 0.22, *p* < 0.001).

#### Neutral vs. Erotic Stimuli–Slides and Clips Conditions

When contrasting neutral and erotic stimuli, neural generators were localized in the left secondary visual cortex (MNI: X = –15, Y = –90, Z = 15; Broadmann area 18) during the static slides (*t* = 0.279, *p* < 0.001) and in the right primary/secondary visual cortex (MNI: X = 15, Y = –85, Z = 15; Broadmann area 17/18) during the dynamic condition (*t* = 0.355, *p* < 0.001). As previously reported for the comparison with fear stimuli also here neutral stimuli, both static slides and dynamic clips, elicited higher Alpha compared to emotional erotic stimuli (see Figure 4B).

The further analysis of Mu rhythm showed the same MNI coordinates for both slides (MNI: X = –15, Y = –90, Z = 15; Broadmann area 18; *t* = 0.24, *p* < 0.001) and clips (MNI: X = 15, Y = –85, Z = 15; Broadmann area 17/18; *t* = 0.35, *p* < 0.001).

#### Fear vs. Erotic Stimuli–Slides and Clips Conditions

sLORETA analysis on emotional categories showed greater Alpha activation in the left precuneus/dorsal posterior cingulate cortex (MNI: X = –10, Y = –45, Z = 50, Broadmann area 7/31) for fear than for erotic slides (*t* = 0.180, *p* < 0.01). When considering the clip condition, fear clips elicited greater Alpha in the left superior parietal lobule (MNI: X = –20, Y = –45, Z = 50, Broadmann area 7) (see Figure 4C).

An additional analysis of Mu rhythm revealed MNI coordinates overlapping those found in Alpha analyses, for both slides (MNI: X = –20, Y = –70, Z = 35; Broadmann area 7; *t* = 0.15, *p* = 0.01) and clips (MNI: X = –15, Y = –50, Z = 45; Broadmann area 7; *t* = 0.32, *p* = 0.005). Unlike the previous contrasts between neutral vs. emotional stimuli (both unpleasant and pleasant), the analysis that directly compared the two emotional stimulations (i.e., fear vs. erotic stimuli) revealed source estimations in left parietal associative areas (left BA 7) for both Alpha and Mu frequencies.

### sLORETA Correlations on Alpha Rhythm and Explicit Measures

As a last analysis we correlated the subjective emotional reports (valence and arousal separately) with the Alpha frequency. The aim was to correlate the two main distinct emotional domains, the subjective and the neurophysiological ones. While arousal ratings were not systematically associated with any Alpha generator, for valence rating two different patterns emerged, depending on the category of stimuli.

#### Neutral Stimuli–Slides and Clips Conditions

**FIGURE 5:**
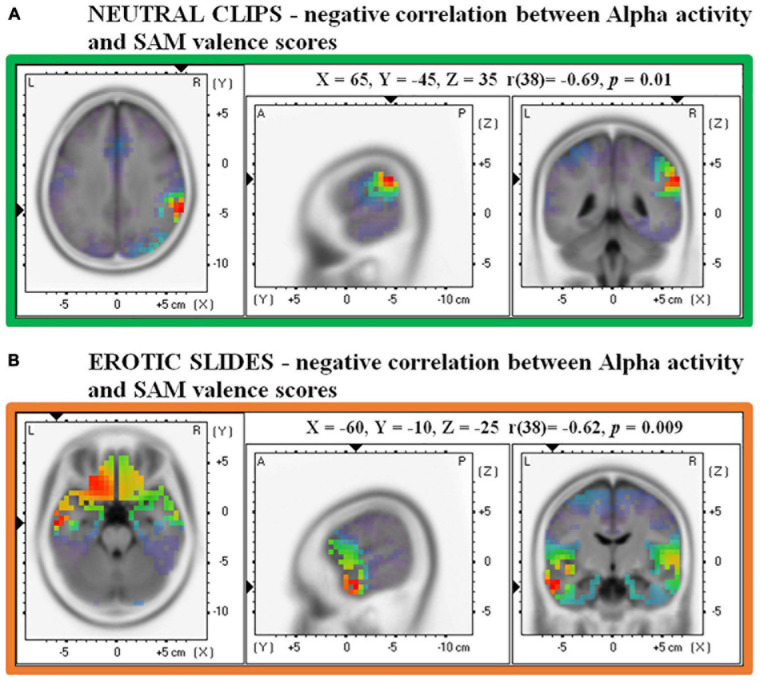
Correlations between source Alpha frequency and valence scores in the SAM. **(A)** Shows a negative correlation for Neutral clips and **(B)** shows a negative correlation for Erotic slides.

Source analysis showed a non-significant negative association between Alpha activity in the right supramarginal gyrus/angular gyrus and valence scores during the presentation of static slides (MNI: X = 65, Y = –45, Z = 35, Broadmann area = 40/39, *r* = –0.57, *p* = 0.07). However, this negative correlation reached statistical significance when dynamic clips were analyzed. Figure 5A shows that lower Alpha in the same right supramarginal area/angular gyrus (MNI: X = 65, Y = –45, Z = 35, Broadmann area = 40/39, *r* = –0.69, *p* = 0.01) was associated with higher valence scores in response to neutral clips.

No significant correlations emerged with arousal measures.

#### Erotic Stimuli–Slides and Clips Conditions

Source analysis on erotic slides revealed that the left middle temporal gyrus (MNI: X =-60, Y = –10, Z = –25, Broadmann area 21) was the brain region which showed the strongest relationship between Alpha activity and valence evaluation (*r* = –0.62, *p* < 0.01). In particular, Figure 5B shows that the lower the Alpha activity in this temporal area, the higher the valence associated with erotic slides. No significant results were found for valence ratings in the clips condition and for arousal ratings in both slides and clips conditions.

No significant correlations emerged between fear stimuli (both slides and clips) and the SAM ratings.

Moreover, no significant correlations emerged between sLORETA and STAI, PANAS, and IRI tests.

## Discussion

The present study investigated the efficacy of different methods of emotional stimulation (i.e., static slides and dynamic movie clips) in the elicitation of pleasant and unpleasant affects. The main goal was to test the hypothesis that, keeping constant some critical variables such as stimulus duration and content, the clips would have elicited larger emotional responses at both subjective and neurophysiological levels. In addition, we aimed to assess the contributions of the left and right hemispheres during positive and negative emotional processing. The EEG Alpha band has been previously demonstrated to be negatively correlated with cortical metabolism measured by PET methods (Oakes et al., 2004) thus, in the present study, the amplitude of EEG Alpha was considered an index inversely related to cortical arousal. Furthermore, as we used images/clips strongly related to action/movement (namely fear, but also erotic induce appetitive action tendency, that is approach toward the pleasant stimulus), the Mu rhythm—typically associated with movement imagery/planning/execution—has been further analyzed. According with the literature (Garakh et al., 2020), the Mu rhythm is larger at fronto-central and in the left parietal sites, whereas Alpha is prevailing at more posterior regions, namely in the parieto-occipital cortices. In the current experiment, the dominant passive setting (participants were forced to stay sitting without moving to avoid artifacts) elicited an Alpha signal peaking in the occipito-parietal regions. Analysis of the Mu band showed a spatial distribution of results and sources overlapped with Alpha, thus supporting the interpretation that the prevailing behavioral setting is immobilization, and the associated dominant band is Alpha. However, the specific activity peaking over somato-sensory cortices could be interpreted consistently also according to the functional meaning of the Mu rhythm in relation to action tendency. Therefore, the greatest Mu suppression in parietal sites can also be interpreted as greater action tendency, the direction of which (moving away or approaching) depends on the motivational program induced by the stimuli (Fear or Erotic) (Bradley and Lang, 2007; Papousek et al., 2009).

All stimuli were evaluated by each participant in terms of arousal and valence. Overall, dynamic stimuli were more effective than static ones in prompting emotional states at subjective level, and this difference was particularly clear for the arousal dimension. Arousal was, in general, higher for clips than for slides, but it was also greater for erotic and fear clips compared to slides while, importantly, no differences emerged in case of neutral stimuli. These results suggest that using dynamic real-to-life materials in emotional settings amplifies both subjective and psychophysiological responses, such as EEG (Gross and Levenson, 1995; Palomba et al., 2000; Schaefer et al., 2010). Moreover, the reliability of dynamic clips was effective both in inducing greater pleasant and unpleasant affects. Our findings extend previous findings which reported that erotic clips were associated with high levels of arousal and fear clips were characterized by low valence and high arousal (Maffei and Angrilli, 2019). Taken as a whole, our data show that dynamic stimuli mainly modulate subjective arousal: although the content of the slides and clips was the same, the exposure to multimodal information (e.g., auditory and visual) and the presence of a more realistic and immersive setting made the participants’ reactions more intense.

The analysis of explicit valence judgments showed the predicted pattern of responses, consisting in higher valence for positive (erotic) than neutral and negative (fear) stimuli, and in lower valence for negative compared to all other stimuli. Both positive and negative emotions elicited the same valence levels regardless of the type of stimulation, whereas the viewing of neutral clips increased the participants’ reported valence in comparison to static slides. Correlations with the empathy scales revealed that participants who reported lower valence for fear clips also had higher empathy scores pertaining to spontaneously adopting other people’s point of view. Interestingly, the influence of empathy on personal emotional reactions is exacerbated by dynamic unpleasant information (Maffei et al., 2019). This suggests that clips are more effective than slides in capturing how individual differences in trait empathy can affect the emotional experience.

As anticipated, our study also aimed to contribute to the still open discussion on the role of the two hemispheres in emotional processing. Indeed, decades of research provided controversial results, some of them supporting the right hemisphere hypothesis (Alves et al., 2009; Bourne, 2010) and others suggesting a different engagement of left and right regions based on emotional valence (Davidson, 1992, 1995). Our source analysis on the Alpha EEG rhythm showed different lateralized patterns for static and dynamic conditions (slides vs. clips). In particular, regardless of the emotional content, the presentation of slides induced greater Alpha power in the left secondary visual cortex in response to neutral than to both erotic and fear stimuli. In contrast, clips elicited greater Alpha power in the homologous right secondary visual cortex again to neutral compared with both erotic and fear contents. Alpha enhancement for neutral materials reflects the inhibitory functional role of this frequency band (Lindsley and Wicke, 1974) and thus indicates that perception of less salient and arousing information results in attenuated cortical responses. Enhanced activation of visual associative areas after emotional stimulation has been widely reported in neuroimaging studies (Lang et al., 1998; Britton et al., 2006; Kale Edmiston et al., 2013). It has been proposed that the amygdala, a key region in the processing of emotionally salient information (Phan et al., 2004; Fitzgerald et al., 2006; Peck et al., 2013), is involved in the modulation of visual cortex activity (Vuilleumier et al., 2004). The mediator role of the amygdala is also supported by anatomical studies on primates that observed projections connecting the amygdala to cortical visual areas in the ventral stream (Amaral et al., 2003). Therefore, this subcortical-cortical pathway could facilitate the perception, and therefore the reaction, to emotional stimuli through a preferential and particularly rapid stimulus processing (Tamietto and De Gelder, 2010). This pattern was further supported by functional data in humans that assessed the coupling of BOLD signal between the amygdala, and the visual system during emotional pictures viewing (Das et al., 2005; Wendt et al., 2011). The visual-emotional circuitry is a good candidate for explaining the occipital activations found in our study. It is worth noting that, consistently with previous data, we observed an increase in Alpha activity for neutral compared to emotional pleasant and unpleasant items (Schubring and Schupp, 2019, 2021). The separate analysis of slides and clips stimuli highlighted a hemispheric asymmetry in the Alpha distribution, characterized by a posterior involvement on the left hemisphere for images and on the right for clips. Given that this result is independent on emotional content (erotic vs. fear), the hemispheric lateralization seems to be induced by the kind of stimulation adopted (slides vs. clips). The right hemisphere activation might be indexing the involvement of the neural circuitry subtending the orienting of visuospatial attention (Hougaard et al., 2015). Moreover, hemisphere specificity in visual areas has also been reported by neuroimaging studies concerning form-specific (right hemisphere) vs. form-abstract (left hemisphere) visual processing (Stevens et al., 2012).

We also estimated the source distribution by contrasting the two emotional categories. Greater Alpha and Mu power in response to fear than to erotic stimuli was found for both slides and clips. Neural generators were localized in the left precuneus/dorsal posterior cingulate cortex during slides presentation and in the left superior parietal lobule during clips presentation. The modulation in this particular brain region suggest that this EEG activity could be interpreted also in line with the functional meaning of the Mu rhythm, that is expected to be inhibited during motor imagery or planning. Indeed, the precuneus plays an important role in visuo-spatial imagery, self-processing operations and, more relevant for this study, in perspective taking and experience of agency (fore review see, Cavanna and Trimble, 2006). Other fMRI findings showed that the attribution of emotions to the self or others activates the left precuneus, the posterior cingulate and the prefrontal areas (Ochsner et al., 2004). In our study, during slides presentation, precuneus activity was always reduced to positive compared to negative stimuli. The relative lower Alpha in visual associative areas to emotional stimuli compared to the neutral ones suggests that high arousing material strengthen visual processing: the lower Mu/Alpha rhythms in left parietal sites can be interpreted in terms of action tendency, greater for emotional compared with neutral stimuli. Furthermore, the differences found between fear and erotic can be interpreted as greater action tendency (motivated approach) to erotic compared with fear (motivated withdrawal) stimuli. Mu/Alpha reduction was also observed in the dorsal portion of the posterior cingulate cortex, a deep region which is recruited during both the evaluation of the affective valence of external stimuli (Maddock and Buonocore, 1997; Maddock et al., 2003) and during moral judgment (Greene et al., 2001). During clips presentation, we observed an increased EEG Mu/Alpha amplitude indicating cortical synchronization (Garakh et al., 2020) for negative compared to positive stimuli in the left superior parietal lobule, a region involved in the integration of somatosensory and visual information, in sensory aspects of motor planning (Caminiti et al., 1996) and in the integration of audio-visual stimuli (Molholm et al., 2006). This result can be further interpreted as a greater recruitment of the dorsal posterior pathway (“where pathway”) starting from the primary visual areas and involved in the processing of dynamic moving images (clips) and location of moving characters in the space, as compared to static images (slides). Concerning our data, we propose that viewing negative stimuli induced a lower engagement of this parietal network compared to positive, highly arousing material.

The Alpha power of the main electrical source was also correlated with explicit image/clip judgments, showing a significant correlation with the valence dimension. More in detail, lower Alpha in the left middle temporal gyrus was associated with greater valence for erotic slides. This result suggests a potential neural correlate of the positive valence dimension. Notably, in our study, participants with smaller inhibition of this region provided higher ratings to pleasant erotic slides. A link between the middle temporal gyrus (but also orbito-frontal areas) and positive valence has been shown in previous fMRI data (Nielen et al., 2009). The responsiveness of the middle temporal gyrus to pleasant images is in line with the functional role of this brain area, in particular with its recruitment during the observation of actions, the processing of words (Papeo et al., 2019) and multimodal semantic processing (Visser et al., 2012). Considering that the presentation time for each picture was long (about 13 s), it is possible that the viewing of pleasant information recruited this left-lateralized region to assign meaning to the emotional content.

Our investigation of the neural correlates of subjective stimulus evaluation also revealed that lower Alpha in the right supramarginal/angular gyrus was associated with greater valence levels during neutral clips. A similar, albeit not significant, negative correlation was also found for the static (slides) condition. This result has two implications: on one hand, we can assume that the dynamic relative to static modality is more sensitive in modulating neural responses; on the other hand, the fact that the same brain area emerged both for pictures and clips reveals a possible neural mechanism involved in the valence evaluation of stimuli with no emotional content (i.e., neutral ones). While the angular gyrus has been associated with a variety of cognitive processes (Seghier, 2012), one of its main roles is the integration of multisensorial information to form a coherent representation of events (Damasio, 1989; Mesulam, 1998), a mechanism that might be also activated during the processing of dynamic clips.

Surprisingly, we did not find correlations between EEG spectral activity and subjective valence/arousal to fear stimuli. The lack of association between cortical activity and subjective evaluations may depend on the lack of variability in behavioral indices, especially when static slides were presented on the screen. At the same time, it may also be due to the characteristics of this type of stimuli. Indeed, threatening clips usually appear with a very dark environment, where the context does not always help to discriminate against the elements present in the scene. A further critical aspect is directly associated with the key role that the soundtrack plays on the threatening scene: indeed, as film directors well know, without audio, a thriller scene loses most of its impact. As a final remark, in many of our threatening stimuli a man and a woman were present on the scene, but almost invariably the predestinate *victim* was the latter. The viewer’s attention was then attracted by the female figure present in the scene. This feature may have fostered identification in our female participants, but not in males. Future research could consider possible gender biases, especially when cognitive and complex emotional stimuli are administered.

Another aspect to consider is that fear situations (i.e., threatening experiences) are less common in everyday life and participants were probably less familiar with this kind of stimuli compared to other ones. However, this is a direct consequence of studying both unpleasant and pleasant states in laboratory settings. Moreover, albeit being more infrequent, stimuli that depict direct threats are nevertheless biologically relevant, especially if participants identify themselves with the victim, due to sharing the same gender or other personal factors. Fear stimuli are also characterized by scenes implying movement, while neutral stimuli do not necessary involve action. However, this was an intrinsic characteristic of the stimuli used for eliciting negative emotions (i.e., threatening). More in general, when stimuli that depict faces are shown, such as was the case for our emotional stimuli, studies have shown that participants attend to faces more than to other parts of the scene (e.g., Hershler and Hochstein, 2005), both during static and during dynamic stimulus presentation. Adding movement slightly reduces the frequency, but slightly increases the duration of the fixations on faces (Stoesz and Jakobson, 2014). This aspect could be further investigated in future studies including neutral stimuli representing movement.

A limitation of this work is that it did not include other physiological indices usually recorded in research on emotions (such as skin conductance or heart rate). Given the informative role of these measures for emotional investigates, future research should consider recording them during the experimental stimulation.

Overall, our research contributed to demonstrate the effectiveness of dynamic, multimodal stimuli in prompting emotional responses at both subjective and physiological levels. Our electrophysiological findings showed a distinct contribution of the secondary visual cortexes of the two hemispheres during the emotional processing of static (left) vs. dynamic (right) information. Correlations with behavioral measures revealed an asymmetric Alpha distribution associated with the subjective rating of neutral and pleasant emotional stimulation. Our findings suggest an important role for oscillatory Alpha activity during emotional experiences.

## Data Availability Statement

The raw data supporting the conclusions of this article will be made available by the authors, without undue reservation.

## Ethics Statement

The studies involving human participants were reviewed and approved by the Ethical Committee for the Psychological Research of the University of Padova (protocol n. 3886). The participants provided their written informed consent to participate in this study.

## Author Contributions

CS and AA conceived to the study. FF and LS implemented the experimental task. ZR collected the data. CS and ZR performed the analyses. ZR and FF drafted the manuscript. ZR, FF, MB, AA, and CS revised the manuscript. All authors approved the final version of the work and agreed to be accountable for all aspects of the work, in ensuring that questions related to the accuracy and integrity of any part of the work are appropriately investigated and resolved.

## Conflict of Interest

The authors declare that the research was conducted in the absence of any commercial or financial relationships that could be construed as a potential conflict of interest.

## Publisher’s Note

All claims expressed in this article are solely those of the authors and do not necessarily represent those of their affiliated organizations, or those of the publisher, the editors and the reviewers. Any product that may be evaluated in this article, or claim that may be made by its manufacturer, is not guaranteed or endorsed by the publisher.
